# Clinical characteristics of ICU patients with gastrointestinal colonisation by *Candida auris*

**DOI:** 10.3389/fcimb.2026.1944762

**Published:** 2026-10-23

**Authors:** Lianghao Fang, Yan Duan, Donghao Cai, Chuangchuang Mei, Xiaojing Hong, Xiaojun Li

**Affiliations:** 1 Department of Intensive Care Medicine, Guangdong Provincial Second Hospital of Traditional Chinese Medicine, Guangzhou, Guangdong, China; 2 Guangdong Provincial Key Laboratory of Research and Development in Traditional Chinese Medicine, Guangzhou, Guangdong, China; 3 Administration Department of Nosocomial Infection, Guangdong Provincial Second Hospital of Traditional Chinese Medicine, Guangzhou, Guangdong, China; 4 Clinical Laboratory, Guangdong Provincial Second Hospital of Traditional Chinese Medicine, Guangzhou, Guangdong, China

**Keywords:** Candida auris, gastrointestinal colonisation, intensive care unit, nomogram, predictive model

## Abstract

**Objective:**

This study aimed to analyse the detection of *Candida auris* in faecal cultures of intensive care unit (ICU) patients and its impact on clinical outcomes, whilst constructing a predictive model for the risk of intestinal carriage of this fungus to inform clinical screening and infection control strategies.

**Methods:**

A retrospective study design was employed, enrolling 970 eligible patients admitted to the ICU of Guangdong Provincial Second Hospital of Traditional Chinese Medicine between January 2024 and December 2025. Participants were categorised into a *C.auris*-positive group (n=13) and a negative group (n=957) based on stool culture results. Data were collected on patients’ underlying medical conditions, antibiotic use and invasive procedures prior to stool culture, treatment-related data and clinical outcome measures. Univariate analysis compared clinical characteristics between groups. Least absolute shrinkage and selection operator (LASSO)regression screened potential predictors, establishing a multivariate logistic regression model visualised as a nomogram. Model performance was assessed via area under the receiver operating characteristic curve (AUC), calibration curves, and decision curves.

**Results:**

Among 970 patients, the detection rate of *C.auris* in stool cultures was 1.34% (13/970). Univariate analysis revealed that the prevalence of anaemia and a history of antifungal drug exposure, as well as the duration of broad-spectrum antibiotic use, were all significantly higher in the positive group than in the negative group. Furthermore, the duration of antimicrobial use and length of hospital stay were both longer in the positive group. Anaemia and a history of exposure to antifungal drugs were independent predictors of intestinal *C.auris* carriage. After incorporating these two factors into the predictive model, the AUC value was 0.828. Calibration curves demonstrated good agreement between predicted and actual detection rates, whilst decision curves indicated clinical net benefit within the 0–15% risk threshold.

**Conclusion:**

The presence of *C.auris* in the faeces of patients in ICU is associated with anaemia, a history of exposure to antifungal drugs. The predictive model developed in this study demonstrates good discriminatory performance, offering a practical means of implementing targeted screening and infection control measures in clinical practice.

## 1 Introduction

*C.auris*, an emerging multidrug-resistant fungal pathogen, was first isolated in 2009 from the external ear canal of an inpatient in Japan. Since then, it has been detected and reported in more than 50 countries and regions worldwide, demonstrating a trend of rapid dissemination and epidemic potential (Satoh et al., 2009; Lionakis and Chowdhary, 2024; Bing et al., 2025). This pathogen not only exhibits high resistance rates to commonly used antifungal agents such as fluconazole but also possesses multiple clinically important characteristics: microscopically, it presents ovoid-to-ellipsoidal budding yeast cells, and rarely forms true hyphae or pseudohyphae under routine culture conditions; it can survive for weeks to months on both biotic and abiotic surfaces, favouring efficient nosocomial cross-transmission (Centers for Disease Control and Prevention, 2024; Santana et al., 2024). Its major virulence-related mechanisms include biofilm formation on medical devices, secretion of aspartic proteases and phospholipases for nutrient acquisition and tissue damage, as well as the capacity to partially evade host innate-immune neutrophil killing (Phan-Canh et al., 2026). Invasive bloodstream infection due to *C.auris* is associated with reported crude in-hospital mortality rates ranging from 30 %-72 % in critically-ill populations (Kim et al., 2024). It poses a severe threat to high-risk healthcare settings such as intensive care units (ICU). Multiple healthcare institutions and ICUs have now reported outbreaks of C. auris infections (Schelenz et al., 2016; Bing et al., 2024; Kappel et al., 2024; Meletiadis et al., 2024), attracting significant attention from public health and medical communities.

The gastrointestinal tract serves as the primary ecological niche for *C.auris* colonisation (Proctor et al., 2021; Meletiadis et al., 2024). This colonisation status constitutes both a significant risk factor for subsequent invasive infection and a critical reservoir for nosocomial cross-transmission and outbreak propagation. Furthermore, reports indicate that 25% of colonised critically ill patients progress to candidemia, resulting in severe adverse outcomes (Lionakis and Chowdhary, 2024). ICU patients, characterised by prolonged hospitalisation, compromised immunity, frequent invasive procedures, and widespread broad-spectrum antimicrobial use, constitute a high-risk cohort for *C.auris* colonisation and infection (Schelenz et al., 2016; Bing et al., 2024; Kappel et al., 2024; Lionakis and Chowdhary, 2024; Meletiadis et al., 2024). In recent years, *C.auris* infection cases in China have exhibited rapid growth, with new cases increasing by over 300% between 2023 and 2024 (Bing et al., 2025). The majority of these cases are concentrated in ICU settings, making ICUs high-risk departments for *C.auris* outbreaks (Sayeed et al., 2020; Alshamrani et al., 2021).

Although existing studies have identified certain risk factors, specific predictive indicators for intestinal colonisation remain unclear. Traditional culture methods suffer from limitations such as time-consuming procedures and insufficient sensitivity, failing to meet the demands for rapid screening and intervention. Developing predictive models based on readily accessible clinical indicators holds significant importance for achieving precise screening of high-risk patients for *C.auris* intestinal colonisation and optimising the allocation of infection control resources. This study focuses on the detection of *C.auris* in ICU patients’ stool cultures, systematically analysing its clinical determinants and association with healthcare outcomes. By identifying key predictors to establish a practical predictive tool, it aims to provide scientific evidence for early clinical identification of high-risk patients and implementation of targeted prevention measures, thereby reducing the risk of nosocomial transmission and the incidence of adverse patient outcomes.

## 2 Materials and methods

### 2.1 Patient data

This is a single-centre, retrospective observational study. All patients admitted to the intensive care unit at Guangdong Second Hospital of Traditional Chinese Medicine between January 2024 and December 2025 were monitored (n = 1 074). All patients underwent routine faecal examination and microscopic examination of faecal smears (1 578 total tests). Stool fungal culture (total = 1 641 specimens, test-level count, including repeated samples from individual patients) was performed for patients presenting with diarrhoea, abnormal stool consistency, fungal detection on microscopic examination of faecal smears, or where the attending physician deemed it clinically necessary. A patient was considered *C. auris*-positive if any one of their stool cultures yielded *C. auris*. Following screening according to inclusion and exclusion criteria, 970 unique patients were finally enrolled and divided into a positive group (n = 13) and a negative group (n = 957).

Case inclusion criteria: all patients admitted to the ICU of Guangdong Second Hospital of Traditional Chinese Medicine between January 2024 and December 2025;

Exclusion criteria were as follows:

1. Age < 18 years;
2. ICU stay < 24 hours;
3. Inability to obtain a valid stool sample post-admission;
4. Known history of *C.auris* colonisation or infection prior to ICU admission;
5. Repeated admissions.

Although all patients undergo stool testing on admission, we do not have systematic stool culture data for asymptomatic patients. This may result in some positive cases being missed, leading to bias in the identification of certain carrier characteristics, and may also underestimate the true prevalence of gastrointestinal *C.auris* carriage amongst ICU patients at this healthcare facility. The research process is illustrated in **Figure 1**. The research received approval from the Ethics Committee of Guangdong Provincial Second Hospital of Traditional Chinese Medicine (Approval No: K202511-007-01).

**Figure 1:**
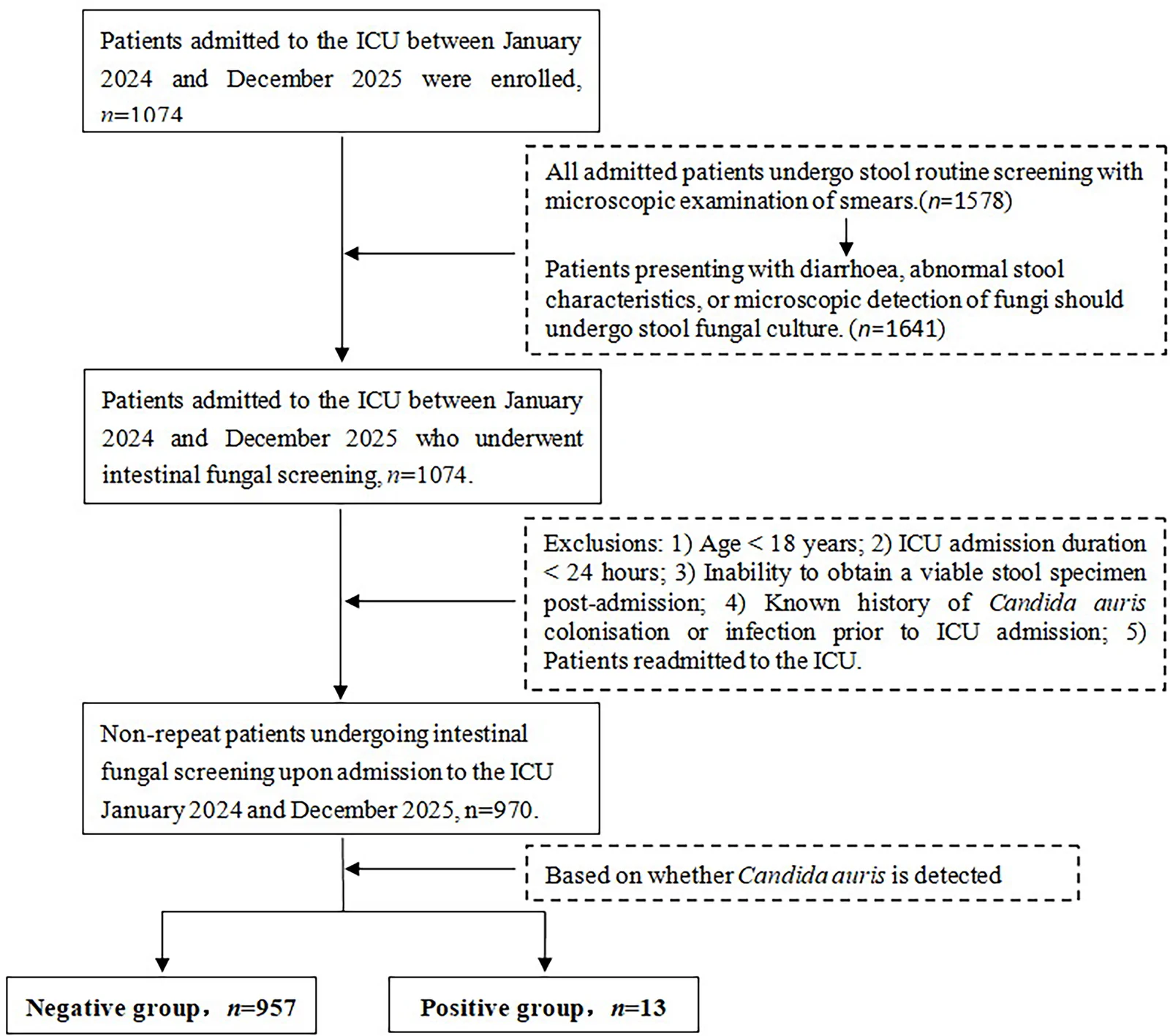
Flowchart for gastrointestinal *C.auris* carriage screening in ICU patients.

### 2.2 Microbiological tests

Routine stool examinations were performed using a fully automated faecal analyser (Wovent FA160, China); Gram-stained smears (Beso Bio, Zhuhai, China) were examined under an optical microscope (Olympus CX-31, Japan). Laboratory identification of *C.auris* was carried out using MALDI-TOF mass spectrometry (Zhongyuan Bio, Chongqing, China, model EXS3000). Antimicrobial susceptibility testing was performed using ATB FUNFUS 3,susceptibility test strips from bioMérieux (France). Susceptibility breakpoints were based on guidelines provided by the US CDC and the European Committee on Antimicrobial Susceptibility Testing: fluconazole ≥32 µg/mL, amphotericin B ≥2 µg/mL (Meletiadis et al., 2024), 5-flucytosine ≥0.5 µg/mL (Centers for Disease Control and Prevention, 2024; European Committee on Antimicrobial Susceptibility Testing, 2024).

### 2.3 Data collection

The following demographic and clinical data were collected: age, gender; underlying conditions at admission, including concomitant renal insufficiency, hepatic insufficiency, electrolyte imbalance, anaemia, hypoalbuminaemia, gastrointestinal dysfunction, cerebral haemorrhage, heart failure, diabetes mellitus, hypertension, cerebral infarction, pneumonia, and Acute Physiology and Chronic Health Evaluation II (APACHE II) score; Prior to the stool culture, data were collected on whether a peripherally inserted central venous catheters (PICC) had been used, whether a urinary catheter (CAU) had been used, whether mechanical ventilation (MV) had been administered, whether antifungal medication had been used, whether total parenteral nutrition (TPN),and the number of days on broad-spectrum antibiotics therapy (DOT). Antibiotic usage post-admission was recorded, including duration of use and types of antibiotics administered. Outcome data were concurrently collected, encompassing length of hospital stay, total hospital costs, and discharge status.

### 2.4 Statistical analysis

Continuous variables meeting normal distribution were expressed as mean ± standard deviation; non-normally distributed data were presented as median (interquartile range). Categorical variables were denoted as n (%). Categorical variables underwent chi-square or Fisher’s exact tests, whilst continuous variables were analysed using t-tests or rank-sum tests. Within the dataset, multiple variable analysis employed the Lasso (Least Absolute Shrinkage and Selection Operator) logistic regression to screen independent risk factors and construct grouping prediction maps. Model performance was assessed via receiver operating characteristic (ROC) curves and calibration curves, with ROC area under the curve (AUC) ranging from 0.5 (no discriminatory power) to 1 (complete discriminatory power). Decision curve analysis (DCA) was concurrently employed to determine the net benefit threshold for predictions. Results with p-values < 0.05 were considered statistically significant. All statistical analyses were performed using R software (version 4.3.1) and MSTATA software (www.mstata.com).

## 3 Results

### 3.1 Baseline data and fungal isolation results of ICU patients

During 2024-2025, 1,074 ICU admissions were recorded, with 1,578 stool routine examinations performed via microscopic examination and 1,641 stool specimens subjected to fungal culture. Following exclusion criteria, 970 patients ultimately met requirements. Fungal isolation occurred in 171 cases, with the predominant isolates being *Candida glabrata* (38.0%, 65/171), *Candida tropicalis* (26.9%, 46/171), *Candida albicans* (15.8%, 27/171), and *C.auris* (7.6%, 13/171). Further fungal isolation details are presented in **Table 1**.

**Table 1:** Distribution of gut-isolated fungi in ICU patients.

Num	Pathogens	Quantity	Percentage
1	*Candida glabrata*	65	38.0%
2	*Candida tropicalis*	46	26.9%
3	*Candida albicans*	27	15.8%
4	*C.auris*	13	7.6%
5	*Candida parapsilosis*	9	5.3%
6	*Candida krusei*	6	3.5%
7	*Saccharomyces cerevisiae*	4	2.3%
8	*Candida portoricanus*	1	0.6%
9	Total	171	100.0%

### 3.2 Analysis of antimicrobial susceptibility test results

As the primary objective of the study was to screen for *C.auris* carriage in the gut, only 8 of the 13 *C.auris* isolates were ultimately subjected to antimicrobial susceptibility testing. Of the five patients who were not tested, two had already been discharged by the time *C.auris* was detected; as for the other three, the clinicians considered that antibiotic susceptibility testing was not necessary. Analysis of the susceptibility test results indicated that the eight *C.auris* isolates from the gut exhibited the highest resistance rate to fluconazole, at 87.5% (7/8); the resistance rate to 5-fluorocytosine was 25.0%, whilst all isolates were susceptible to amphotericin B. The specific results are presented in **Table 2**.

**Table 2:** Antimicrobial susceptibility results for *C.auris*.

Antifungal drug	Susceptible	Intermediate	Resistance
5-Fluorocytosine,n(%)	5(62.5%)	1(12.8%)	2(25.0%)
Amphotericin B,n(%)	8(100.0%)	0(0.0%)	0(0.0%)
Fluconazole,n(%)	0(0.0%)	1(12.5%)	7(87.5%)

### 3.3 Results of univariate analysis

The data from 970 patients were categorised into a negative group (n=957) and a positive group (n=13) based on *C.auris* detection status. Univariate analysis was conducted on age, gender, APACHE II scores, and underlying comorbidities between the two groups (**Table 3**). The results showed that the median age in the negative group was 73 years, the proportion of males was 63.2 per cent, and the median APACHE II score was 20 points; there were no statistically significant differences in any of these three variables compared with the positive group (all P-values were greater than 0.05). Regarding comorbidities, no statistically significant differences were observed between groups for renal insufficiency (*p* = 0.251) or hepatic insufficiency (*p* = 0.741). However, a significant difference in anaemia prevalence was observed between groups: 76.9% in the positive group versus 25.2% in the negative group (*p* < 0.001). Furthermore, prior to faecal culture, the number of patients on mechanical ventilation, the proportion receiving antifungal medication, and the duration of broad-spectrum antibiotic use were all higher in the positive group than in the negative group, with all differences being statistically significant (*p* < 0.05).

**Table 3:** Univariate comparison of two groups of ICU patients.

Characteristic	Negative group *n* = 957	Positive group *n* = 13	Statistic^*^	*p*-value
age, Median (Q1, Q3)	73 (62, 82)	72 (62, 78)	0.311	0.757
male, n (%)^*^	605 (63.2%)	6 (46.2%)	0.954	0.329
APACHE-II, Median (Q1, Q3)	20 (16, 26)	24 (16, 30)	0.723	0.470
Invasive procedures				
PICC	631 (65.9%)	12 (92.3%)	2.900	0.089
CAU	629 (65.7%)	11 (84.6%)	1.284	0.257
**MV**	**231 (24.1%)**	**8 (61.5%)**	**7.753**	**0.005**
**Antifungal**	**80 (8.4%)**	**7 (53.8%)**	**27.170**	**<0.001**
**DOT**	**5 (3, 7)**	**7 (4, 14)**	**2.175**	**0.030**
TPN, n (%)	226 (23.6%)	2 (15.4%)	0.134	0.714
Underlying conditions				
Cerebral haemorrhage, n (%)	95 (9.9%)	1 (7.7%)	0.040	0.842
Cerebral infarction, n (%)	319 (33.3%)	6 (46.2%)	0.458	0.498
Hypertension, n (%)	608 (63.5%)	8 (61.5%)	0.020	0.887
Heart failure, n (%)	259 (27.1%)	7 (53.8%)	3.375	0.066
Pneumonia, n (%)	570 (59.6%)	11 (84.6%)	3.352	0.067
Renal insufficiency, n (%)	353 (36.9%)	7 (53.8%)	0.938	0.333
Hepatic insufficiency, n (%)	200 (20.9%)	3 (23.1%)	0.023	0.880
Gastrointestinal insufficiency, n (%)	222 (23.2%)	4 (30.8%)	0.097	0.756
Diabetes mellitus, n (%)	348 (36.4%)	7 (53.8%)	1.020	0.313
Electrolyte imbalance, n (%)	105 (11.0%)	2 (15.4%)	0.003	0.953
**Anaemia, n (%)**	**241 (25.2%)**	**10 (76.9%)**	**15.305**	**<0.001**
Hypoproteinaemia, n (%)	150 (15.7%)	4 (30.8%)	1.204	0.273

APACHE II, Acute Physiology and Chronic Health Evaluation II;PICC, Peripherally Inserted Central Venous Catheters;CAU,urinary catheter; MV,mechanical ventilation; DOT, days of antibiotics therapy; TPN,total parenteral nutrition. Bold values indicate P < 0.05.

^*^ The Statistic column presents calculated test values: χ² for χ² test, Fisher’s exact statistic, *Z*-value for rank-sum test, or *t*-value for *t*-test according to data distribution.

^**^ n (%): The number of positive cases in each group for this variable and the proportion of the total in that group.

### 3.4 Impact on clinical outcomes

Compared with patients in the negative group, detection of *C.auris* was associated with increased duration of antibiotic use and significantly prolonged hospital stays. No statistically significant differences were observed in the number of antibiotics used, total hospital costs, or discharge outcomes (**Table 4**).

**Table 4:** Clinical outcomes among hospitalised patients carrying *C.auris*.

Characteristic	Negative group *n* = 957	Positive group *n* = 13	Statistic^*^	*p*-value
**Duration of antibiotic use, Median (Q1, Q3)**	**18 (12, 26)**	**26 (17, 36)**	**2.030**	**0.042**
Antifungal agents, n (%)	574 (60.0%)	8 (61.5%)	0.012	0.909
β-lactams, n (%)	845 (88.3%)	12 (92.3%)	0.001	0.990
Carbapenems, n (%)	537 (56.1%)	8 (61.5%)	0.153	0.695
Glycopeptides, n (%)	376 (39.3%)	7 (53.8%)	1.137	0.286
Quinolones, n (%)	339 (35.4%)	3 (23.1%)	0.401	0.527
Tetracyclines, n (%)	163 (17.0%)	5 (38.5%)	2.753	0.097
**Polymyxin, n (%)**	**54 (5.6%)**	**3 (23.1%)**		**0.036** ^**^
**Length of concurrent hospital stay, Median (Q1, Q3)**	**6 (2, 12)**	**16 (10, 24)**	**3.441**	**<0.001**
Total cost, Median (Q1, Q3)	82,807 (54,754, 124,987)	96,251 (82,292, 150,150)	1.406	0.160
Discharge status, n (%)	136 (14.2%)	1 (7.7%)	0.073	0.788

^*^ The Statistic column presents calculated test values: χ² for χ² test, Fisher’s exact statistic, *Z*-value for rank-sum test, or *t*-value for *t*-test according to data distribution.

^**^ Fisher’s exact test. Bold values indicate P < 0.05.

### 3.5 Development of patient carriage risk model

#### 3.5.1 Variable selection

21 variables including age, gender, APACHE II score, and underlying comorbidities, whether an invasive procedure was performed prior to stool culture, and whether antifungal medication was used, amongst others across both groups were incorporated into the initial model. Using LASSO regression analysis, the model was simplified to two potential predictors: anaemia and whether antifungal medication had been taken prior to the stool culture. The cross-validation error plot for the LASSO regression model is presented in **Figure 2**.

**Figure 2:**
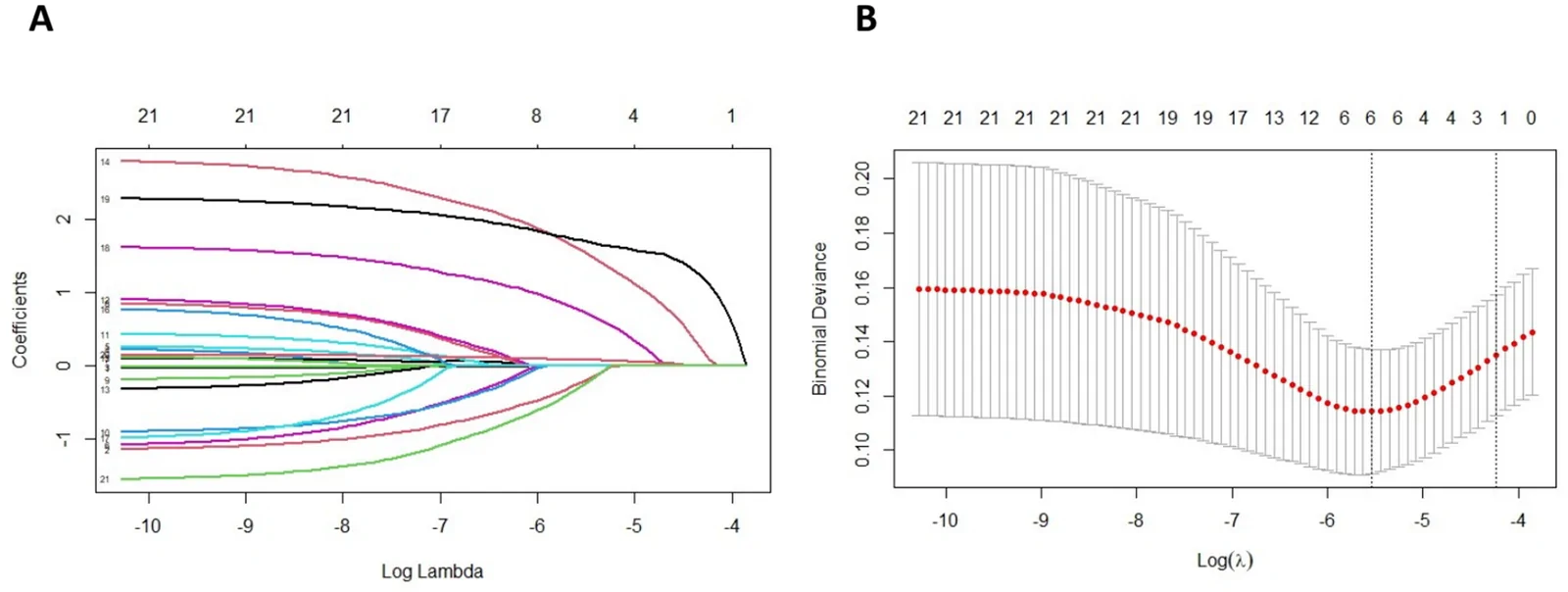
Cross-validation error plot of the LASSO regression model. **(A)** LASSO regression coefficient path plot; **(B)** LASSO regression cross_validation plot.

#### 3.5.2 Multivariate regression analysis

A multivariate regression model was constructed using the two variables selected via LASSO regression; anaemia and the use of antifungal drugs were both independent predictors of *C.auris* carriage in the faeces of ICU patients (**Table 5**); The odds ratio (OR) for anaemia was 7.784 (95% confidence interval [CI]: 2.081 to 29.119, *p* = 0.002); the OR for the use of antifungal drugs was 9.687 (95% CI: 3.099 to 30.287, *p* < 0.001).

**Table 5:** Multivariate analysis of *C.auris* carriage at admission.

Characteristic	OR	95% CI	*p*-value
Anaemia	7.784	2.081, 29.119	0.002
Antifungal	9.687	3.099, 30.287	<0.001

OR, odds ratio; CI, confidence interval.

#### 3.5.3 Constructing a nomogram

The final logistic regression model comprised two independent predictors and was developed into a user-friendly predictive nomogram, as illustrated in **Figure 3**. The associated risk increased with the presence of underlying anaemia, antifungal.

**Figure 3:**
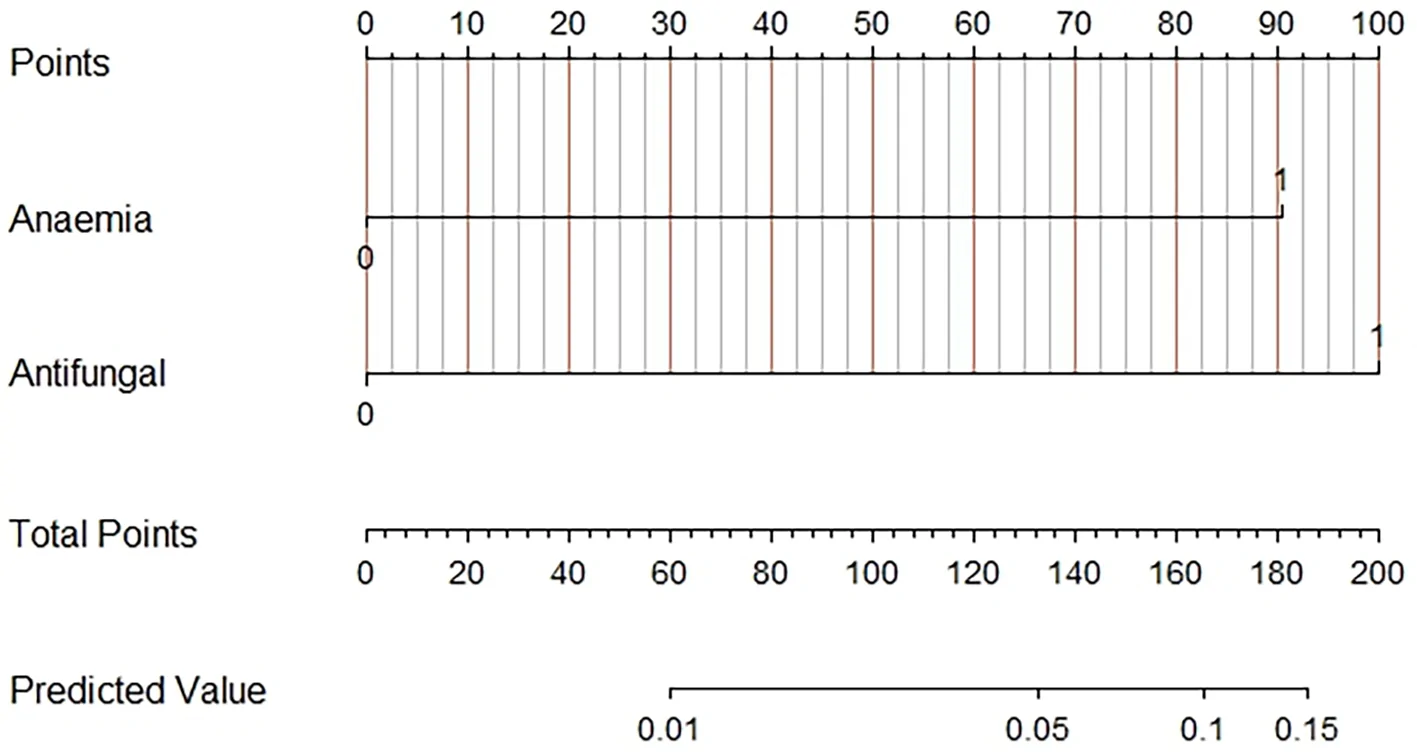
Nomogram of the prediction model.

#### 3.5.4 Model validation

##### 3.5.4.1 Model AUC value

Receiver Operating Characteristic (ROC) curve analysis indicates that this predictive model possesses strong discriminatory power, with an area under the curve (AUC) of 0.828; the AUC for anaemia was 0.727, and for antifungal was 0.759. Refer to **Figure 4** for further details.

**Figure 4:**
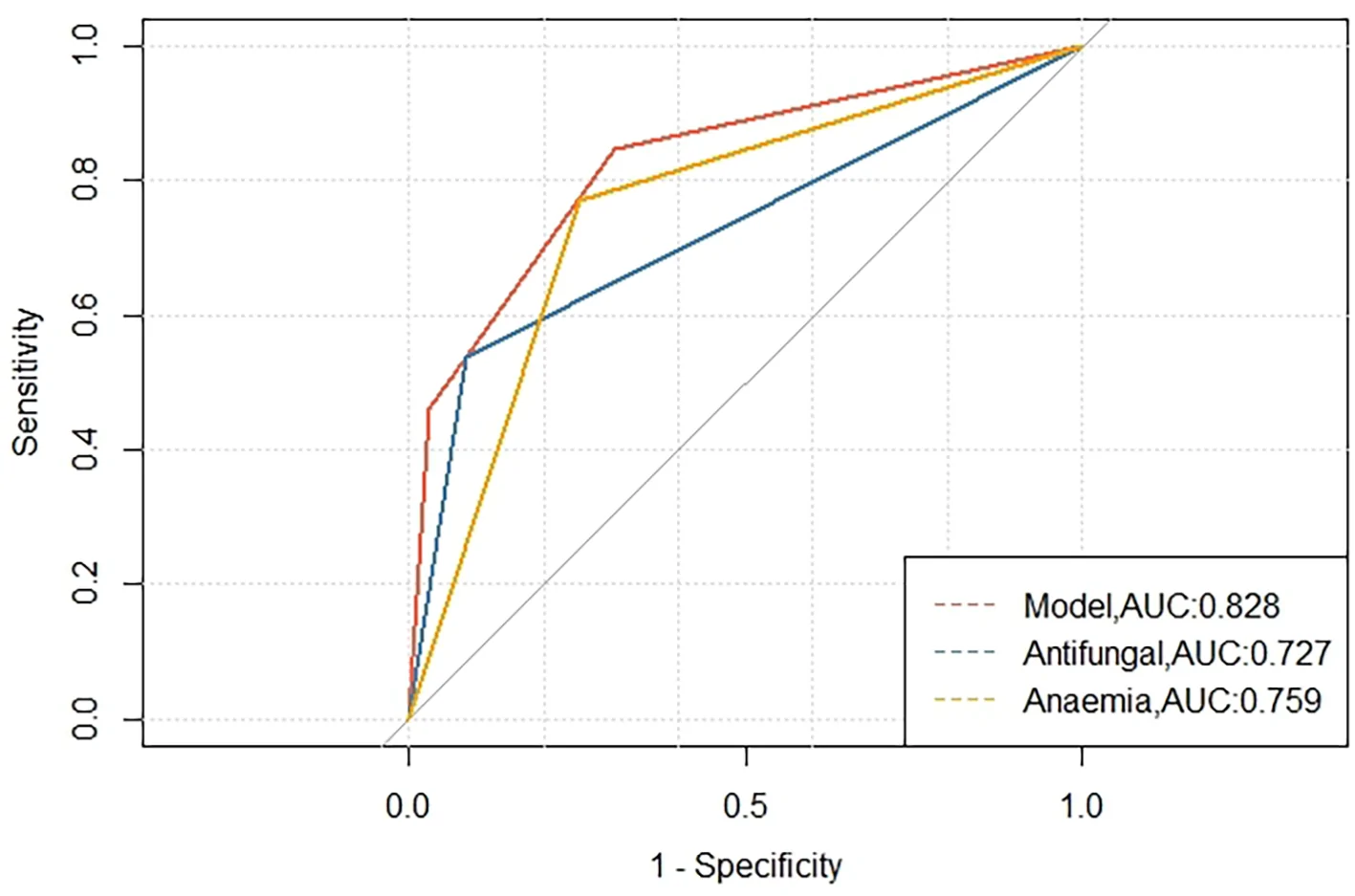
Prediction model and AUC for each variable.

##### 3.5.4.2 Calibration curve of the prediction model

The calibration plot for this prediction model is shown in **Figure 5**. The results indicate a strong correlation between the observed and predicted groups. Validation findings demonstrate that the original prediction model remains valid on the validation dataset, with its calibration curve closely approximating the ideal curve. This confirms that the predicted outcomes align with actual findings.

**Figure 5:**
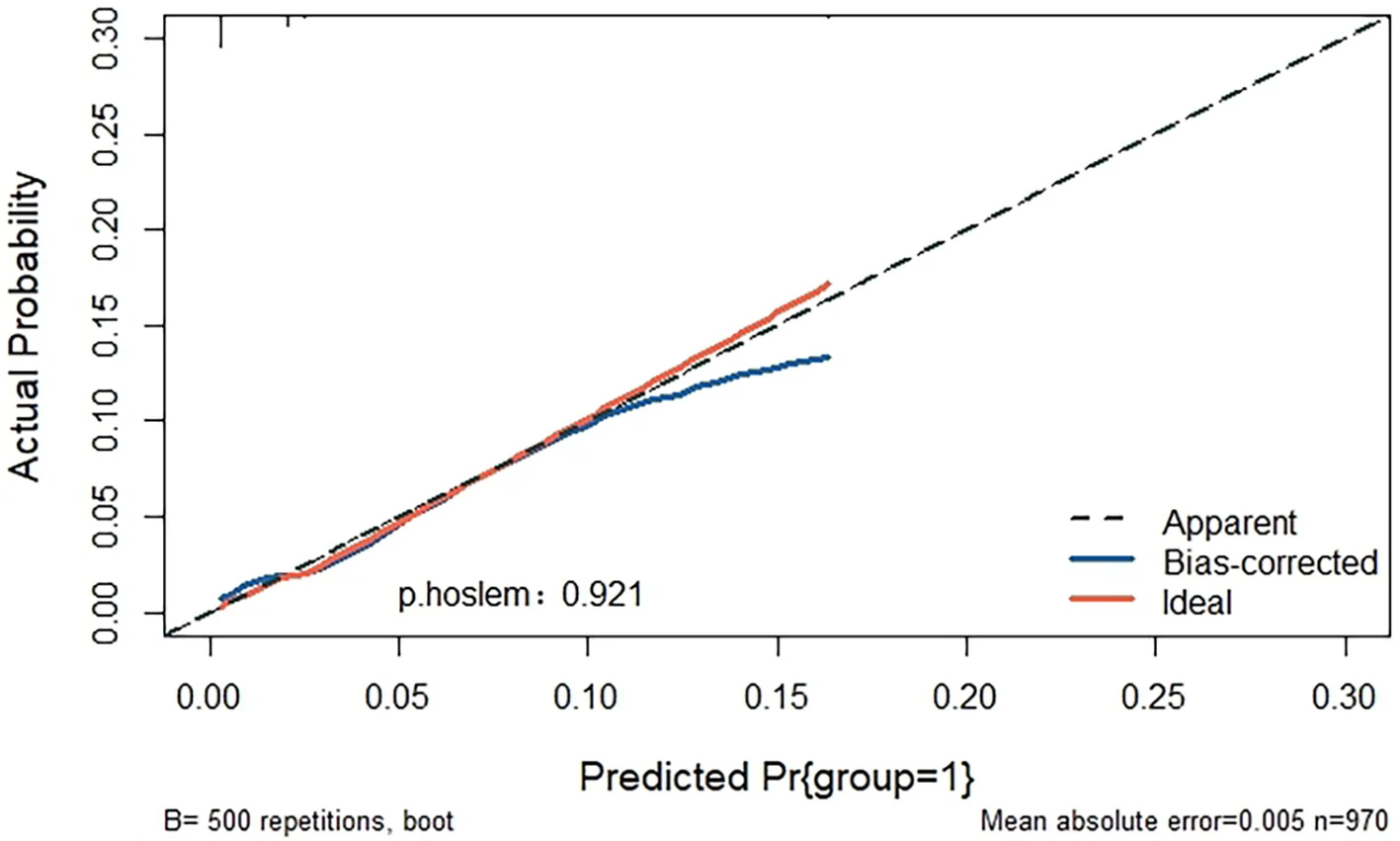
Calibration curve of the predictive model.

##### 3.5.4.3 Decision curve analysis

**Figure 6** presents the DCA with this prediction model. Results indicate that intervention within the 0–15% risk threshold zone yields clinical benefit compared to no intervention.

**Figure 6:**
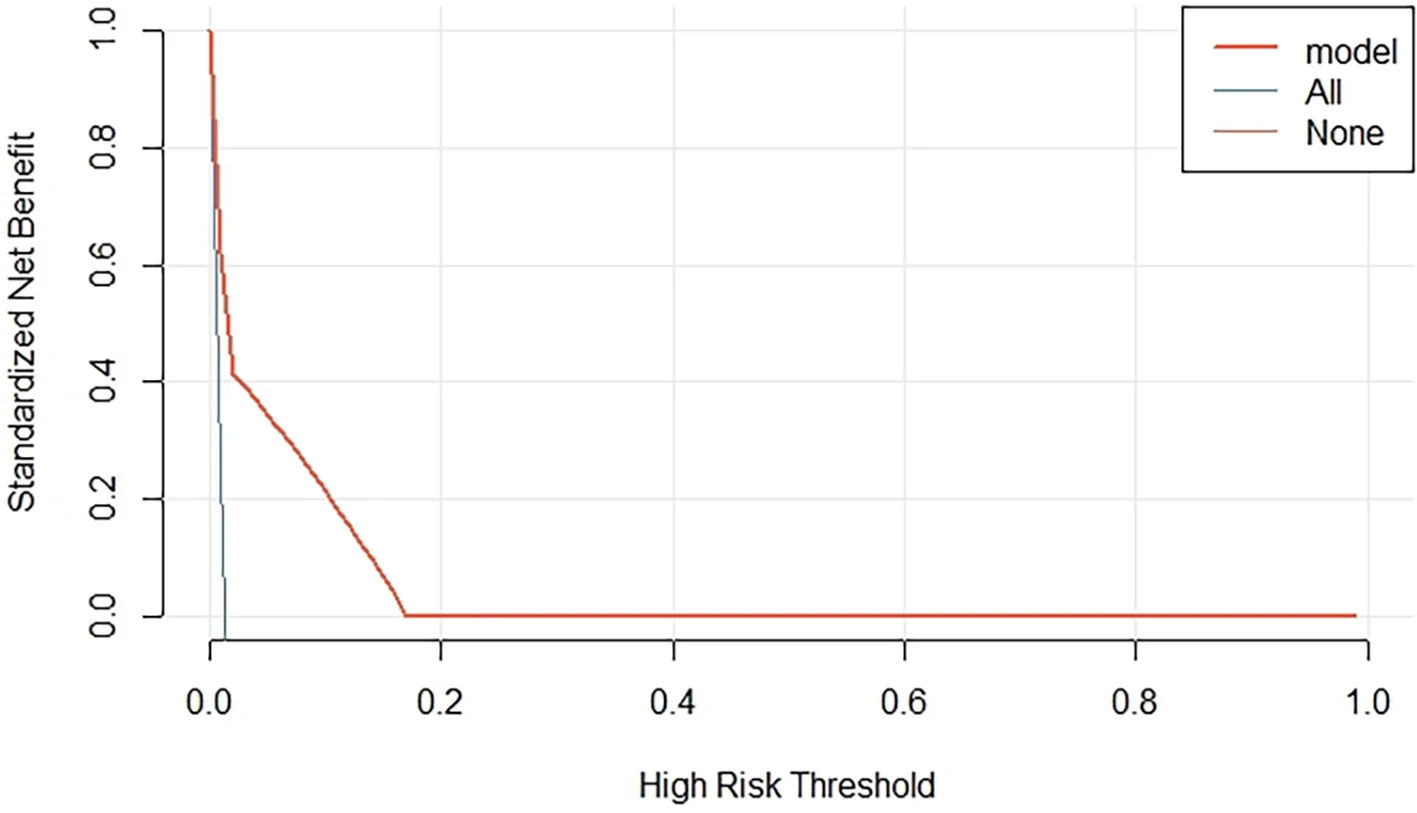
Clinical decision curve for the predictive model.

## 4 Discussion

This study focused on the clinical characteristics and risk prediction of *C.auris* gastrointestinal colonisation in ICU patients. It established anaemia as an independent predictor of intestinal colonisation and demonstrated that the constructed nomogram model possesses good discriminatory performance. Furthermore, it revealed that colonisation prolongs both antimicrobial usage duration and hospital stay, providing crucial evidence and practical tools for the precise prevention and control of *C.auris* within intensive care settings.

The detection rate of *C.auris* in stool cultures amongst ICU patients was 1.34% (13/970), accounting for 7.6% of intestinal fungal isolates. This proportion was lower than that of *Candida glabrata* (38.0%) and *Candida tropicalis* (26.9%), aligning with reported levels of *C.auris* constituting 11.2% of clinical *Candida* isolates (Chen and Feng, 2025). C*.auris* colonisation enables rapid fungal dissemination within patients. Notably, colonised patients exhibited a significantly higher median duration of antimicrobial use compared to non-colonised patients, alongside a nearly threefold increase in hospital stay duration relative to the control group. *C.auris* colonisation may influence clinical outcomes through two pathways: firstly, colonisation status indicates intestinal dysbiosis, necessitating prolonged antimicrobial regimens to cover potential infection risks; Secondly, clinical studies indicate (Lockhart et al., 2017; Tharp et al., 2023; Saunders et al., 2026) that *C.auris* may persist on the skin for months or even over a year due to the difficulty of eradicating it from the skin surface. Consequently, the cautious clinical management of colonised patients may indirectly prolong hospital stays. Although this study found no statistically significant association between colonisation and total hospital costs or discharge outcomes, excessive antimicrobial use may exacerbate antimicrobial resistance pressures and increase the risk of subsequent invasive infections. High-risk patients, such as those with catheter or other medical device insertions, prolonged intensive care unit stays, or prior surgeries, face heightened susceptibility to invasive fungal infections (Lockhart et al., 2017; Shastri et al., 2020; Saunders et al., 2026). Research indicates that patients with *C.auris* colonisation are more susceptible to candidemia (Ruiz-Gaitán et al., 2018), which carries a mortality rate exceeding 30% once established (Chakrabarti et al., 2015; Chen et al., 2020; Benedict et al., 2023), resulting in severe adverse outcomes. Notably, none of the 13 patients detected with *C.auris* colonisation in this study subsequently developed candidemia. This discrepancy may stem from differences in population distribution. In addition, pathogenicity variations amongst distinct *C. auris* strains could also modify the likelihood of progression from gastrointestinal colonisation to invasive bloodstream infection. Further genomic and phenotypic investigations are required to verify this hypothesis, which warrants longer-term observation.

The study found that the use and duration of antibiotic treatment were key factors influencing the presence of *C.auris* in ICU patients’ faeces. The results of the univariate analysis identified an increased duration of broad-spectrum antibiotic use and a history of exposure to antifungal drugs as key risk factors for gastrointestinal colonisation with *C. auris* in ICU patients. Prolonged exposure to broad-spectrum antibiotics eradicates commensal anaerobic bacteria such as *Bacteroides* and *Firmicutes* indiscriminately. These bacterial communities maintain the gut’s resistance to fungal colonisation by secreting short-chain fatty acids and inducing the intestinal epithelium to secrete the antifungal peptide LL-37 (Samonis et al., 1993; Fan et al., 2015). Following microbial depletion, the protective effects conferred by commensal bacteria are eliminated. This includes physical support for intestinal mucosal integrity, short-chain fatty acid secretion, as well as induction of host epithelial cells to produce antifungal peptide LL-37. The gut mycobiome may also participate in regulating *C. auris* colonisation via fungus-fungus interactions. Resident intestinal fungi could compete for nutritional substrates or release antifungal metabolites to restrain *C. auris* overgrowth. Broad-spectrum antibacterial and antifungal agents may disrupt not only bacterial communities but also the native gut mycobiome, potentially removing inter-fungal inhibitory pressure and facilitating *C. auris* establishment (Zhai et al., 2020; Huang et al., 2024; Janeczko and Skrzypek, 2025).Collectively, these changes reshape the intestinal nutrient and carbon source microenvironment and create a favourable niche for the proliferation and persistent colonisation of *C.auris (*Rather et al., 2022).Conversely, ICU patients often receive empirical antifungal therapy for suspected or confirmed invasive fungal infections. Repeated exposure to drugs continuously selects for *C.auris* clones that carry ERG11 gene mutations and have upregulated TAC1B efflux pumps (Carolus et al., 2021; Doorley et al., 2023; Li et al., 2023). This enhances the survival advantage of these strains within the gut. Among the eight *C.auris* strains isolated, 87.5 per cent were resistant to fluconazole. Previous reports (Sankaran and Weiss, 2015; Weiss et al., 2019; Elstrott et al., 2020; Zhu et al., 2020; Duan et al., 2025) indicate that *C.auris* exhibits a higher fluconazole resistance rate than other *Candida* species. However, the resistance rate observed in this study is higher than that currently reported. This may be due to disruption to the homeostasis of the gut microbiota caused by the use and duration of antibiotics, as well as prior exposure to antifungal agents. This has led to the survival of *C. auris* that is resistant to antifungals and the isolation of highly resistant strains.

Multivariate regression analysis revealed anaemia to be a strong independent predictor of *C.auris* intestinal colonisation, with a significantly higher prevalence of 76.9% in the anaemic group compared to 25.2% in the non-anaemic group. This finding supplements existing knowledge on risk factors for *C.auris* colonisation. Previous studies have predominantly focused on factors such as immunosuppression and invasive procedures (Lockhart et al., 2017; Shastri et al., 2020; Saunders et al., 2026). This research, however, is the first to establish a strong association between anaemia and intestinal colonisation. The underlying mechanisms may involve three aspects: first off, anaemia frequently coexists with underlying conditions such as chronic kidney disease and malnutrition, which inherently compromise host defence functions (Sankaran and Weiss, 2015); Furthermore, iron metabolism disorders may compromise intestinal mucosal integrity, creating conditions conducive to fungal colonisation—iron being an essential element for microbial growth, altered iron utilisation efficiency in anaemic states may foster a microenvironment favourable for *C.auris* proliferation (Elstrott et al., 2020). Lastly, anaemia may serve as a marker of systemic inflammation, where inflammatory responses disrupt gut microbiota balance and reduce resistance to pathogen colonisation (Weiss et al., 2019).

The predictive model developed in this study, incorporating anaemia, a history of antifungal drug exposure, achieved an AUC of 0.828. Calibration curves demonstrated good agreement between predicted and actual colonisation rates. Decision curves indicated a net clinical benefit within the 0–15% risk threshold, confirming the model’s reliable discriminatory capacity and practical utility. From a clinical practice perspective, this model offers significant advantages: firstly, all predictors are readily accessible routine indicators at ICU admission, requiring no additional testing and meeting rapid risk assessment needs; secondly, the visual design of the nomogram facilitates clinician operation—for instance, in the case of a patients with anaemia and a history of antifungal medication exposure, the model’s total score can be used to rapidly estimate the probability of colonisation, thereby determining the priority for stool culture or PCR testing (Hennessee et al., 2024); Thirdly, it optimises screening resource allocation. Model-based targeted screening reduces unnecessary testing within risk threshold ranges, enhancing resource efficiency. Early screening coupled with timely antimicrobial stewardship may constitute effective control factors for preventing *C.auris* colonisation and infection (Lockhart et al., 2017; Huang et al., 2021; Rossow et al., 2021). For instance, Jimenez et al. (2026) implemented a screening questionnaire within electronic medical records for hospitalised patients, successfully identifying colonised individuals. This facilitated early implementation of infection prevention and control measures, effectively preventing horizontal transmission of *C.auris* within healthcare settings.

This study has certain limitations: To start with, its single-centre retrospective design with a relatively limited sample size in the positive group may affect the statistical power for variables such as heart failure and pneumonia, necessitating multi-centre validation for the model’s generalisability; In addition, it did not differentiate between types of anaemia, which may exert varying effects on colonisation risk. As a final point, it did not track long-term outcomes for colonised patients, such as progression to invasive infection or recurrence of infection. Future research may proceed along three avenues: In the first place,conducting multicentre prospective studies to expand sample sizes and incorporate additional potential predictors, thereby optimising model performance; Furthermore, deepening exploration of the pathophysiological mechanisms linking anaemia to *C.auris* colonisation, such as validating the impact of iron metabolism on fungal proliferation through *in vitro* experiments; Ultimately, evaluate the efficacy of targeted prevention strategies guided by the model, such as implementing early isolation and decolonisation therapy for high-risk patients as proposed by Chiara et al (de Groot et al., 2026), to determine whether this reduces nosocomial transmission rates and the incidence of invasive infections. Furthermore, integrating rapid molecular diagnostic technologies with this predictive model to establish an integrated workflow of risk assessment-rapid detection-precision prevention may further enhance *C.auris* control in ICUs. Moving forward, drawing on the experience gained from this study, we intend to collaborate more effectively with other healthcare institutions to conduct multicentre and prospective studies to further validate the causal relationship.

In summary, this study confirms that anaemia and a history of antifungal drug exposure are key independent predictors of gastrointestinal colonisation with *C.auris* in ICU patients. The constructed nomogram effectively identifies high-risk individuals, providing a practical tool for targeted screening and infection control in clinical practice. *C.auris* colonisation prolongs antimicrobial use and hospital stays, necessitating enhanced monitoring and management of high-risk patients to mitigate adverse outcomes.

## Data Availability

The raw data supporting the conclusions of this article will be made available by the authors, without undue reservation.
